# High-Performance Adhesive Joint Made from Densified Wood

**DOI:** 10.3390/polym14030515

**Published:** 2022-01-27

**Authors:** Benjama Meethaworn, Suthon Srivaro, Sureurg Khongtong

**Affiliations:** 1 Materials Science and Engineering Program, School of Engineering and Technology, Walailak University, Thasala District, Nakhon Si Thammarat 80160, Thailand; benjama1559@gmail.com (B.M.); ssuthon@wu.ac.th (S.S.); 2 Petrochemical and Polymer Engineering Program, School of Engineering and Technology, Walailak University, Thasala District, Nakhon Si Thammarat 80160, Thailand; 3 Center of Excellence on Wood Science and Biomaterials, Walailak University, Thasala District, Nakhon Si Thammarat 80160, Thailand; 4 Center of Excellence on Petrochemical and Materials Technology, Chulalongkorn University, Bangkok 10330, Thailand

**Keywords:** adhesive joint, densified wood, polymer impregnation

## Abstract

The strength of an adhesive joint plays a major role in the implementation of engineering wood products; therefore, joint performance receives intense scrutiny. This study investigated a wooden adhesive joint, made from densified wood, the performance of which was dramatically enhanced. The wood sample was developed by performing mechanical compression and polymer impregnation on rubberwood. This treated rubberwood was additionally prepared by simple surface sanding prior to jointing. The highest wettability was found on surfaces sanded with the largest grit sandpaper. Consequently, glueline thickness increased with progressively larger grit (smaller grit number) sandpaper. In addition, the maximum shear strength for the joint made from the densified rubberwood was greater than that of that made from the original one, by 40%. Surprisingly, with the optimal sanding treatment, the shear strength of the wooden joint gradually increased with an increase in the density of the densified rubberwood from 1.05 to 1.30 g/cm^3^. Moreover, the rate of wood failure also increased throughout the stated density range.

## 1. Introduction

Wood is one of the most important sources of natural polymer used globally. The rubber tree (Hevea brasiliensis) has become economically significant in farm forestry for natural rubber; in fact, more than 80% of wooden furniture products are comprehensively manufactured from rubber trees when they are aged and become low in latex yield [1]. However, the structural application of rubberwood is still restricted due to its limited mechanical properties and durability, compared with those of typical structural materials [2]. The impregnation of rubberwood using methyl methacrylate and mixtures of glycidyl methacrylate and styrene, followed by their polymerization, improves the water absorption, dimensional stability, degradability, tensile strength, flexural strength, and hardness of the modified samples [3,4,5]. Additionally, this makes rubberwood more resistant to natural degradation. Recently, high-strength rubberwood has been successfully developed by using a combination of mechanical compression and polymer impregnation processes [6]. It was reported that this densified wood bore the potential for structural application. Therefore, the adhesive jointing of this wood should be explored in order to provide an opportunity to use densified rubberwood for wider engineering applications. 

The quality of adhesive wooden joints is essentially affected by both the wood and adhesive properties, such as the wood density, strength of the wood tissue, grain angle, surface roughness, adhesive thickness, and adhesive viscosity [7,8,9,10]. However, wood is a porous material; the mechanical interlocking between adhesives and wood microstructure is anticipated to be the main mechanism determining the strength of an adhesive joint [11,12,13]. Thus, for high-density wood species typically selected for structural engineering wood products, the mechanism of interlocking might be limited, as the thick cell walls and small lumens cause a low degree of penetration of the adhesive into the microstructure at the interphase of wood adherends. Studies on adhesive joints with different types of adhesives prepared from wood samples, the surfaces of which were physically and chemically treated by various methods, were performed by many researchers, in order to improve the performance of a joint made of high-density wood species, but a pronounced improvement was not observed [7,14,15].

The main objective of this study was to investigate the optimum adhesive jointing of relatively high-density rubberwood that had been successfully manufactured by our research group [6]. Surface sanding with abrasives of varying roughness was performed on wood samples prior to jointing in order to optimize the levels of surface microrupture for the samples [16]. This procedure was expected to enhance the mechanism of interlocking at the interface of adhesive and rubberwood. In addition, a stronger and denser wood structure, generated by the densification process, was also presumed to enhance the degree of interlocking [6]. The properties at the joints made of the densified samples, compressed with three compression ratios of 0%, 30%, and 50%, compared with those made from the original rubberwood, are presented herein.

## 2. Materials and Methods

### 2.1. Preparation of Densified Rubberwood

Rubberwood lumber with a 12% moisture content and dimensions of 300 mm (longitudinal) × 27 mm (tangential) × 50 mm (radial) was prepared for the densification process. The preparation of the densified rubberwood was performed following the densifying processes proposed previously [6]. The pre-compression of the rubberwood was firstly conducted using a hydraulic press. The wood sample was compressed in the radial direction with three different targeted compression ratios (CR) of 0%, 30%, and 50% using pairs of steel bars with the desired thickness under a constant clamping pressure of 130 kg/cm^2^ (pressure gauge). After the wood sample was compressed to the desired compression ratio, the fixation was maintained for 3 s, and the press was then opened. After that, all the wood samples were dried at 103 ± 2 °C using a hot air oven until a constant weight was attained.

The dry, pre-compressed samples were then subjected to a methyl methacrylate impregnation process. This process was conducted under vacuum (pressure = −0.1 mPa) in a pressure container. The pressurization of a methyl methacrylate monomer (MMA) solution (98 wt% MMA mixed with 2 wt% benzoyl peroxide) in such an environment was performed under an air pressure of 6 kg/cm^2^ for 60 min. The MMA-impregnated wood samples were then removed from the container and wrapped in aluminum foil. They were consequently transferred to steel mounting and compressed at 90 °C to the same targeted thickness as performed in the previous step, for 90 min. The polymerization of polymethyl methacrylate (PMMA) was finalized when the samples were put into a hot air oven at 90 ± 2 °C for 22.5 h, and, consequently, the monomer solution turned to a solid polymer embedded inside the wood structure. Then, the aluminum foil was removed from the sample, and the sample was immediately dried at 103 ± 2 °C for another 24 h until a constant weight was attained.

During the densification process, the values of the monomer uptake (*MU*) during the impregnation process (calculated from Equation (1)), polymer loading (*PL*) after densification process (calculated from Equation (2)), and densities of the original rubberwood and densified rubberwood at 0% moisture content (calculated from Equation (3)) were also recorded.

(1) $$MU\left(\%\right)=\frac{m_{i}-m_{0}}{m_{0}}\times 100$$

(2) $$PL\left(\%\right)=\frac{m_{Poly}-m_{0}}{m_{0}}\times 100$$

(3) $$\rho_{0}=\frac{m_{0}}{V_{0}}$$

where *m_i_* = the weight of the MMA-impregnated wood samples, *m*_0_ = the weight of the original or pre-compressed rubberwood with a 0% moisture content, *m_Poly_* = the dried weight of the densified sample, and *V*_0_ = the volume of the original or pre-compressed rubberwood with a 0% moisture content.

### 2.2. Adhesive Joint Preparation

After densification, all samples were equilibrated in a conditioning room at 20 °C and relative humidity of 65% until a constant weight was attained. These samples were then cut into specimens for surface preparation with dimensions of 75 mm (longitudinal) × 25 mm (tangential) × 10 mm (radial). The tangential–longitudinal surfaces of the wood samples were subjected to sanding with a grinding-polishing machine (Buehler, Germany) at a speed of 200 rpm for 30 s. Sandpapers with grit numbers of 40, 60, 80, and 100 were selected to introduce various levels of microdamage to the surfaces of the specimens. After mechanical abrasion, the residual particles were removed from the abraded surface using a soft brush.

Polyurethane adhesive (PU)—GSP PU 902H and GSP PU 902—supplied by GSP Products, Thailand, was selected for this study, in order to fulfill the ranges for the engineering applications of wood joints [17,18,19]. A mixture of this commercial PU adhesive with a ratio of GSP PU 902H to GSP PU 902 of 1:3 was prepared at ambient temperature. The chemical types and some properties of this composition are shown in Table 1. The adhesive mixture (250 g/m^2^) was applied onto the tangential–longitudinal surface of the stated specimen with a hand brush, and a primer was not present, in order to minimize the complexity of the system. The glued specimens were then placed into contact with one another to form the adhesive joint, and it was laid in a pressing machine at room temperature. A pair of steel bars with a thickness of about 18 mm were also placed in a pressing machine as stoppers, to provide the pressing distance to those joints, and a pressing time of 300 min was allowed. After that, all assembled joints were kept in a conditioning room at 20 °C and relative humidity of 65% for 1 week before further use.

### 2.3. Monitoring of Properties

#### 2.3.1. Contact Angle

A specimen cut from the rubberwood that had been equilibrated under a temperature of 20 °C and 65% relative humidity was used in this study. Contact angle measurement on the tangential–longitudinal surfaces (20 cm × 20 cm) of the specimens was performed using a goniometer (Drop Master, DM300) with the instrument’s software (FAMA). A sessile drop (6 µL) of distilled water was applied, and the contact angle was taken immediately after a drop of water was applied for 5 s. Each reported value was the average of 10 measurements on 10 samples. A two-way analysis of variance, at 0.05 level of significance, was used to examine the effect of the surface sanding (from 40- to 100-grit sanding) on contact angle values of the densified rubberwood with various compression ratios.

#### 2.3.2. Scanning Electron Microscopy

Microscopic images of the cut specimen, with dimensions of about 5 mm (tangential) × 5 mm (radial) × 2 mm (longitudinal), from the sanded sample or the glueline region of the adhesive joint were monitored by scanning electron microscopy with an FEI Quanta 400 SEM (Hillsborom, OR, USA). An accelerating voltage of 10 kV was used to acquire the images. The examined surfaces of the specimens were coated with a thin layer of gold using a sputter coater (Cressington sputter coater 108Auto, Watford, UK) and then mounted on aluminum stubs before performing the tests.

#### 2.3.3. Glueline Thickness

The measurement of the glueline thickness was conducted on the cross-sectional planes of glueline regions of specimens with dimensions of 10 mm × 20 mm using an optical microscope (ME600, Nikon, Tokyo, Japan), according to the method reported previously [10]. The reported value was evaluated from five different points of each glueline from 10 samples.

#### 2.3.4. Block Shear Strength

The block shear strength was measured for the cut specimens from the adhesive joint, with the glued dimensions of 25 mm × 25 mm, using a universal testing machine (UTM, Lloyd LR 150R UTM, Hampshire, UK) [20]. The specimen was loaded in tangential–longitudinal planes with a crosshead speed of 2 mm/min. The block shear strength (τ) was then calculated using the following equation:

(4) $$\tau =\frac{V_{\max}}{A_{s}}$$

where *V*_max_ = the maximum shear force, and *A_s_* = the shear plane area of the specimen.

## 3. Results and Discussion

### 3.1. Densification Process for Rubberwood

In order to prepare the densified rubberwood sample, sawn dried lumber was treated according to three-stage procedures—namely, the pre-compression of the rubberwood, impregnation of methyl methacrylate (MMA), and mechanical compression of the MMA-impregnated rubberwood. The stated procedure was crucial in order to produce the densified rubberwood, as we reported previously [6]. The values of the monomer uptake (*MU*), polymer loading (*PL*), and density (*ρ*_0_) with a 0% moisture content of the densified rubberwood, displayed in Table 2, were compared with those of the original sample. In general, the *MU* and *PL* decreased but *ρ*_0_ increased with an increase in compression ratio, a relationship already discussed. It should also be noted that the smaller percentages of *MU* found in the samples compressed with higher percentages of compression ratios (CRs) might reflect a reduction in production cost as they gained density and strength [6]. 

### 3.2. Topography and Wettability at the Surface of Rubberwood Samples

**Figure 1:**
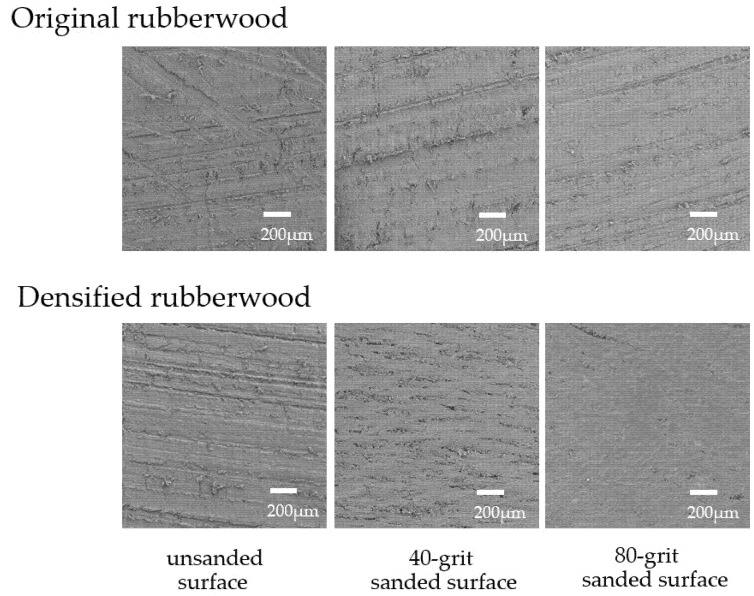
Scanning electron microscope images of the original rubberwood and 50% CR densified one with unsanded and sanded (with 40 and 80 grits) surfaces.

**Figure 2:**
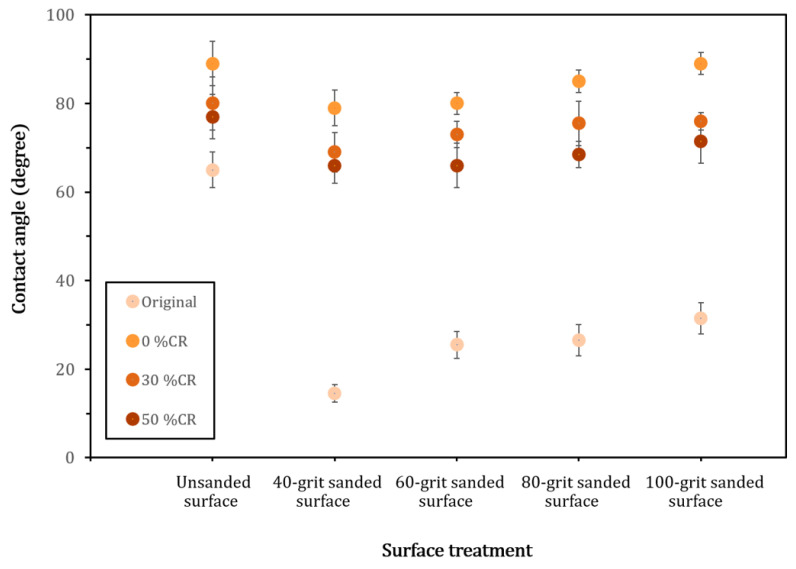
The average values of contact angles on the tangential–longitudinal planes of the original rubberwood and densified rubberwood surfaces. Test specimens were treated with various surface abrasions and compression ratios (CRs). The error bars represented the standard deviations of the data points in this experiment.

Since the surface characteristic of substrates plays a major role in the properties of adhesive joints [8,10,14], the topography of the surface to be glued (longitudinal–tangential plane) of the representative made of the original and the 50% CR densified rubberwood was examined using scanning electron microscopy (SEM). It was shown that mechanical abrasion probably smoothened the surface of both samples. The finer the grits, the smoother the surfaces (Figure 1). In addition, it was demonstrated that there were deposits covering the surface of the tightly packed wood tissue, indicating impregnated PMMA as well as denser wood microstructure in the densified sample. The wettability at the surface of all of the samples used in this study was then monitored by contact angle measurement. Figure 2 shows the values of the contact angles of the original rubberwood, compared with those of the densified one compressed with various CRs of 0%, 30%, and 50%. It was found that the unsanded original samples exhibited higher surface wettability (lower values of contact angles) than the densified one, by about 10 to 20 degrees. This might reflect different levels of hydrophilicity and porosity at the interphases of these samples. This was also consistent with the result reported previously when there was polymer embedded in the wood microstructure, leading to lower hydrophilicity and porosity in the impregnated wood samples [21]. In addition, the contact angles on the non-abraded surfaces of densified samples compressed with various CRs tended to rise with the polymer loading (PL%) in these specimens (shown in Table 1). 

In the same experiment, when the surfaces of the samples were sanded with a 40-grit abrasive, it was found that the new surface of the original rubberwood displayed a drastic drop in the contact angle to 14 degrees (from 66 degrees), while that of the densified samples showed only a slight decrease, from the range of 76–90 degrees to 65–78 degrees, as shown in Figure 2. This might demonstrate that the high contact angle values observed on the surface of the non-abraded sample resulted from the surface portions created by sawing as well as migration of the hydrophobic composition from bulk wood [16,22]. This hydrophobic layer was then eliminated by surface abrasion, resulting in the drastic drop in the contact angle values observed herein. In addition, the abraded surfaces of densified samples yielded lower wettability because PMMA resulted in a greater hydrophobic effect, compared with the original one, thus minimizing the outcome of sanding. Likewise, the samples compressed with lower CRs, containing higher degrees of polymer loading (PL%), exhibited lower surface wettability, as shown in Figure 2.

Typically, it was reported that surface sanding caused a microscopic rupture of wood tissue at the upper surface layer [13,16]. This would encourage surface wettability due to faster penetration of water through the crushed microstructure. Therefore, this effect also contributed to the result for the sanded surface shown in Figure 2. This hypothesis was consistent with the results found for rubberwood surfaces sanded with finer abrasives (higher grit number from 60 to 100 grits); their wettability showed a statistically significant decline (*F*_(3,6)_ = 14.1, *p* < 0.05). The mechanism responsible for this behavior may have been the degree of tissue damage that occurred at the abraded surfaces; the finer the abrasives, the lesser the damage. A similar tendency for contact angle on the sanded surface of rubberwood was reported elsewhere [1].

### 3.3. Characteristic of the Adhesive Joint 

**Figure 3:**
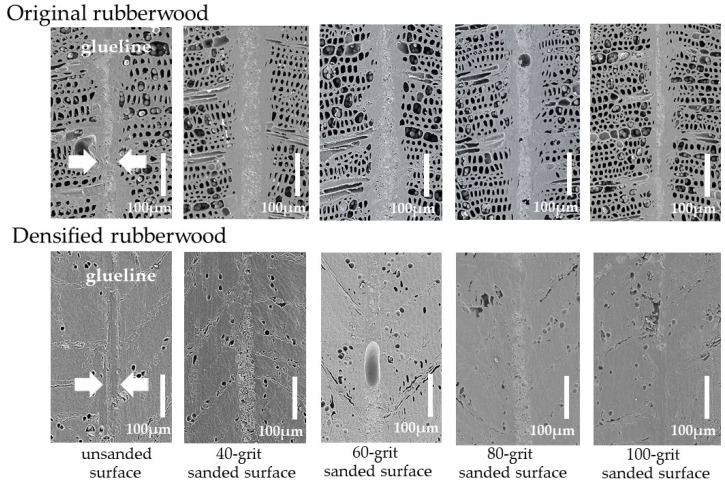
Scanning electron microscope images of glueline region for the joints made from the original and densified rubberwood with unsanded and sanded surfaces.

After the adhesive joint made from rubberwood was prepared, the topography of the adhesive joint was examined using scanning electron microscopy (SEM). Images of the cross-sectional (tangential radius) surface of the adhesive joint from the representatives made of the original and the densified sample with a 50% CR are shown in Figure 3. It was observed that the joint made of the original rubberwood exhibited a more pronounced, thicker glueline region than that made of the densified one. This possibly reflected the effects of compression imposed during jointing; the specimens were compressed from 20 mm to 18 mm, and this led to microscopic damage of wood tissue. Thus, the microstructure of the original wood sample was subjected to more severe damage than that of the reinforced sample. Some of this damage accounted for the formation of glueline observe herein. Additionally, surface sanding with a 40-grit abrasive tended to magnify the boundary of the glueline. This strongly supports the previously suggested consequence of surface abrasion. Chunks of ruptured wood microstructure at the surface of the sanded sample also accounted for the formation of the glueline, and therefore, this sample exhibited a thicker glueline. Comparably, the joint made from wood samples with lower degrees of surface rupture (sanded using finer abrasives, 60–100 grits) displayed thinner gluelines. It was also revealed, as shown in Figure 3, that there was some porosity inside the adhesive band. This was probably due to the CO_2_ generated from the reaction of the isocyanate in the adhesive and the moisture in wood samples. 

**Figure 4:**
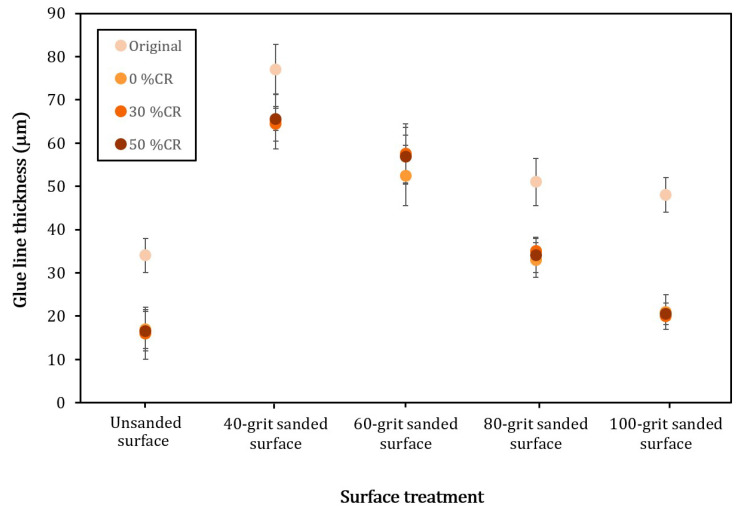
Glueline thicknesses of the original and densified samples with different %CRs. They were also abraded with various grit-number abrasives. The result for the unsanded sample is also included for comparison. The error bars represented the standard deviations of the data points in this experiment.

To investigate the additional influence of surface abrasion on the thickness of the glueline, more adhesive joints of rubberwood samples were monitored by using an optical microscope, and the thickness of the glueline was evaluated. It was found that non-abraded joints intended to have thinner glueline (Figure 4). In addition, the joints made from the original rubberwood displayed thicker gluelines than those made from the densified samples. These results were consistent with those discussed and shown in Figure 3. Furthermore, the thickness values for the densified samples, compressed with various %CRs, were about the same. This might reflect that the degree of tissue damage due to the jointing in the densified sample was roughly in the same range. Then, the surfaces of samples sanded with various abrasives, from 40 to 100 grits, were monitored. It was found that the thickness of the glueline rapidly increased, reaching its peak when the surface was sanded with 40 grits, and then gradually descended after sanding using finer abrasive. These results are also consistent with those shown in Figure 3 and support the formation of the glueline discussed previously. It should also be noted that, at each specific grit number, the sanding of the densified wood surface yielded approximately the same range of glueline thickness. This might indicate that the degree of surface damage due to sanding was about the same for the densified samples. Additionally, the glueline thicknesses for these samples were lower than those found in the joints made from the original wood, and, again, they were consistent with those reported in Figure 3. Therefore, the results might reflect the certain conditions of each particular sample used in this study. 

### 3.4. Shear Strength of the Adhesive Joint

**Figure 5:**
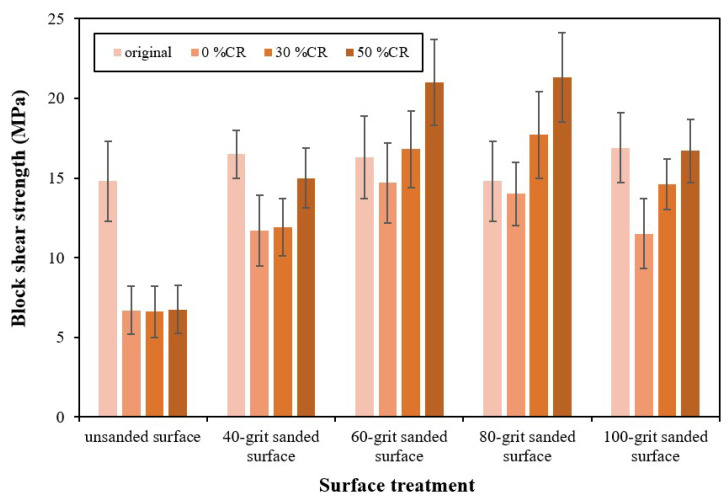
Shear strengths of adhesive joints prepared using various abrasives. The results for the joints made from the original rubberwood and densified samples at different compression ratios (%CRs) are also incorporated. The error bars represented the standard deviations of the data in this experiment.

To investigate the performance of the joint made from densified rubberwood, a block shear strength test was conducted. After shear strength testing, all failed surfaces were examined to determine the types of failure and whether they occurred in the adhesive or wood tissue [13]. The test results for the original rubberwood showed no significant difference in shear strength between the unsanded and sanded samples with 40- to 100-grit abrasives (Figure 5). It should be noted that various levels of porosity were found inside the glueline of the joint made from original rubberwood (as shown in Figure 3). Therefore, the uniformity of shear strength shown here ruled out the effect of porosity on joint performance. It was also found that this joint displayed the same type of failure that occurred within the wood fiber, as shown by the 100% wood failure rates in Table 3.

The experiment performed for the joints made from the densified rubberwood revealed a different tendency. For the non-abraded surface, they showed the lowest value of shear strength. In addition, the values for the densified samples with compression ratios from 0% to 50% were probably in the same range. Moreover, the failure of these joints always occurred in the region of adhesive sitting above wood tissue, as shown by 0% wood failure in Table 3. This might indicate that PMMA-reinforced wood tissue was responsible for the poor performance of the joint from this treatment. 

The joints made from the densified samples with sanded surfaces were then examined, and they exhibited a gradual improvement in shear strength after sanding using finer abrasives, as shown in Figure 5. This was contrary to the results for the wettability and glueline thickness for these treated joints mentioned above. It was also found that using 60- and 80-grit abrasives resulted in peak shear strength, and then, it tended to decline when 100-grit abrasive was used. The peak shear strength, 22 MPa, was about three times greater than that for the non-abraded surface, and this value was about 40% higher than that for the joint made from the original rubberwood. In addition, the maximum shear strength was approximately in the same range as that of the solid densified sample itself reported previously [6]. It appears that the 60–80-grit sanding treatment provided an optimum level of surface abrasion and, therefore, probably, an optimal level of mechanical interlock with the adhesive [11,12]. Additionally, the type of failure shifted to occurring within wood tissue, especially for the jointing of samples with the optimum surface abrasion (Table 3). The optimum surface roughness for the performance of wooden joints was also discussed elsewhere [11,15,23,24]. 

It is very interesting to note that, with the optimal surface sanding (40- and 80-grit), the joints made from densified wood compressed with greater %CRs, resulting in increased densities of 1.05–1.30 g/cm^3^ (Table 2) and, therefore, an increase in shear strength as well as percentages of wood failure. The result found here is contrary to the typical tendency for the bond strength found for joints made of high-density wood, the performance of which decreases when the density is above 0.8 g/cm^3^ [13]. Therefore, this may indicate that the strengthened wood tissue formed from the densification process is responsible for the mechanism of interlocking at the joint made from densified rubberwood. The results shown in this section, as a result, may indicate the achievement of good performance for a wooden joint made from relatively high-density wood. 

## 4. Conclusions

The experiments reported in this paper revealed the effects of compression ratios and surface sanding on the performance of the joint made from densified rubberwood prepared by the method of mechanical compression and polymer impregnation. It was found that the sanding of the surface of rubberwood resulted in an increase in surface wettability and glueline thickness of samples. Surface sanding with finer abrasives, from 40 to 100 grits, however, reduced those properties. 

The value of shear strength for the adhesive joints showed a tendency different from that for the wettability and glueline thickness. The joint made from the densified rubberwood showed an increase in shear strength of about three times after surface abrasion, with 60- and 80-grit sanding, while this effect was not observed for that made from the original wood sample. Moreover, it was also shown that higher compression ratios provided to the rubberwood samples during the densification process resulted in greater shear strength of the joint. This value increased to 22 MPa, 40% greater than that for the joint made from the original rubberwood. Additionally, the percentages of wood failure also increased with the optimal sanding and compression ratios.

## Figures and Tables

**Table 1:** Chemical types and properties of PU adhesive.

Properties	GSP PU 902H	GSP PU 902
Chemical types Appearance	Polyol Gray slurry	Diisocyanate Brown liquid
Density (at 30 °C) g/cm^3^	0.98	1.20
Viscosity (at 30 °C) cps.	700	200

**Table 2:** Comparison of monomer uptake (*MU*), polymer loading (*PL*), and density (*ρ*_0_) of the original and densified rubberwood.

Wood Samples	*MU* (wt%)	*PL* (wt%)	*ρ*_0_ (wt%)
Original rubberwood	-	-	0.69 ± 0.02
Densified rubber wood with 0% CR	67.09 ± 2.83	53.49 ± 4.37	1.05 ± 0.03
Densified rubber wood with 30% CR	54.09 ± 3.06	35.67 ± 2.88	1.22 ± 0.04
Densified rubberwood with 50% CR	43.13 ± 2.81	16.82 ± 1.38	1.30 ± 0.02

**Table 3:** Wood failure percentages.

	Wood Used to Make Joints			
Surface	Original Wood	0% CR Wood	30% CR Wood	50% CR Wood
Non-abraded	100%	0%	0%	0%
40-grit sanded	100%	20%	30%	60%
60-grit sanded	100%	60%	80%	100%
80-grit sanded	100%	60%	90%	100%
100-grit sanded	100%	20%	40%	80%

## Data Availability

All data are available upon reasonable request from the corresponding author.
